# Cardiomyopathies: An Overview

**DOI:** 10.3390/ijms22147722

**Published:** 2021-07-19

**Authors:** Tiziana Ciarambino, Giovanni Menna, Gennaro Sansone, Mauro Giordano

**Affiliations:** 1 Internal Emergency Department, Hospital of Marcianise, 81037 ASL Caserta, Italy; tiziana.ciarambino@gmail.com; 2 Department of Medical Science, University of Campania, L. Vanvitelli, 81100 Naples, Italy; mennagiovqnni@hotmail.com (G.M.); gennaro.sansone90@gmail.com (G.S.)

**Keywords:** cardiomyopathies, sudden cardiac arrest, dilated cardiomyopathy, hypertrophic cardiomyopathy, restrictive cardiomyopathy, arrhythmogenic cardiomyopathy, takotsubo syndrome

## Abstract

Background: Cardiomyopathies are a heterogeneous group of pathologies characterized by structural and functional alterations of the heart. Aims: The purpose of this narrative review is to focus on the most important cardiomyopathies and their epidemiology, diagnosis, and management. Methods: Clinical trials were identified by Pubmed until 30 March 2021. The search keywords were “cardiomyopathies, sudden cardiac arrest, dilated cardiomyopathy (DCM), hypertrophic cardiomyopathy (HCM), restrictive cardiomyopathy, arrhythmogenic cardiomyopathy (ARCV), takotsubo syndrome”. Results: Hypertrophic cardiomyopathy (HCM) is the most common primary cardiomyopathy, with a prevalence of 1:500 persons. Dilated cardiomyopathy (DCM) has a prevalence of 1:2500 and is the leading indication for heart transplantation. Restrictive cardiomyopathy (RCM) is the least common of the major cardiomyopathies, representing 2% to 5% of cases. Arrhythmogenic cardiomyopathy (ARCV) is a pathology characterized by the substitution of the myocardium by fibrofatty tissue. Takotsubo cardiomyopathy is defined as an abrupt onset of left ventricular dysfunction in response to severe emotional or physiologic stress. Conclusion: In particular, it has been reported that HCM is the most important cause of sudden death on the athletic field in the United States. It is needless to say how important it is to know which changes in the heart due to physical activity are normal, and when they are pathological.

## 1. Introduction

Cardiomyopathies are a heterogeneous group of pathologies characterized by structural and functional alterations of the heart [1]. The recently proposed MOGE(S) nosology system embodies all of these characteristics, and describes the morphofunctional phenotype (M), organ(s) involvement (O), genetic inheritance pattern (G), etiological annotation (E), including genetic defect or underlying disease/substrate, and the functional status (S) of the disease using both the American College of Cardiology/American Heart Association stage and New York Heart Association functional class. The proposed nomenclature is supported by a web-assisted application and assists in the description of cardiomyopathy in symptomatic or asymptomatic patients and family members in the context of genetic testing [2]. Dilated cardiomyopathy is one of the main causes of heart failure [3,4]. Hypertrophic cardiomyopathy (HCM) is the most common inherited cardiomyopathy due to mutations in numerous genes. Restrictive cardiomyopathy (RCM) is a heart-muscle disease characterized by stiffness of the ventricular walls leading to diastolic dysfunction, raised end-diastolic pressure, and dilated atria [1] . Arrhythmogenic cardiomyopathy (ARCV) is a pathology characterized by the substitution of the myocardium by fibrofatty tissue, which determines the development of arrhythmias, reduced systolic function, and sudden cardiac death, especially in young patients [1]. Takotsubo cardiomyopathy, also known as stress-induced cardiomyopathy or broken-heart syndrome, is defined as an abrupt onset of left ventricular dysfunction in response to severe emotional or physiologic stress [1]. The purpose of this narrative review is to focus on the most important cardiomyopathies, their epidemiology, their genetic aspects, diagnosis, and their management.

## 2. Methods

Clinical trials were identified by Pubmed until 30 March 2021. The search keywords were “cardiomyopathies, sudden cardiac arrest, dilated cardiomyopathy, hypertrophic cardiomyopathy, restrictive cardiomyopathy, arrhythmogenic cardiomyopathy, takotsubo syndrome”. The studies were selected and their references were reviewed for potential inclusion. Studies written in languages other than English were excluded. Three authors (G.S., G.M., and T.C.) reviewed all study abstracts. All selected studies were qualitatively analyzed.

### 2.1. Dilated Cardiomyopathy (DCM)

Dilated cardiomyopathy is typically characterized by dilatation and impaired function of one or both ventricles. Patients may develop heart failure, and most often the presenting symptoms may be arrhythmias, atrial or ventricular, and sudden cardiac death [3,4]. This disease can be classified as either primary or secondary DCM. Primary DCM is considered idiopathic and the diagnosis can only be made after excluding secondary causes [5].

Dilated cardiomyopathy is one of the main causes of heart failure. Its prevalence, in the population with heart failure, is 1:250–400 patients, in the general population it is 1:2500 patients. The incidence of dilated cardiomyopathy is 7 cases per 100,000 people per year [6,7]. In addition, in the 1990s, idiopathic dilated cardiomyopathy was indicated as the leading cause of heart transplantation in the United States of America [8]. In the pediatric population, dilated cardiomyopathy accounts for 60% of cardiomyopathies, and children under 12 months have the highest incidence [9,10]. A study by Falker et al., published in the NEJM in 2000, analyzed the possible causes of dilated cardiomyopathy in 1230 affected patients and showed these percentages [11].

**Figure 1:**
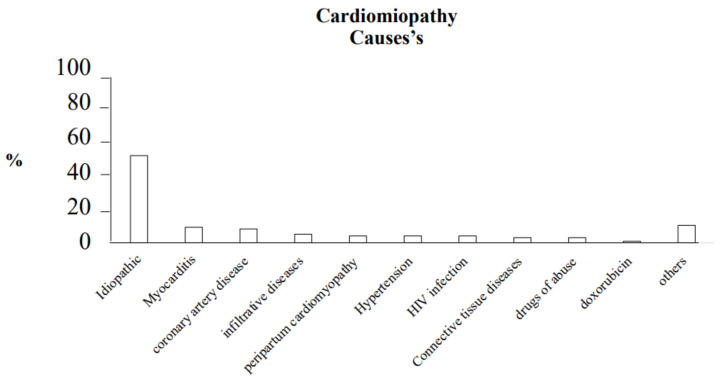
In this figure, we report the most important causes of dilated cardiomyopathy (%).

In Figure 1 we report the most important causes of dilated cardiomyopathy [11].

Among the viruses, those with the greatest myocardial tropism are:

(a) Enteroviruses (coxsackievirus B2);
(b) Adenoviruses;
(c) Parvovirus B19;
(d) Herpesviruses;
(e) Epstein–Barr virus;
(f) Rarely, hepatitis viruses;
(g) Other infectious causes (Lyme disease and Chagas) [6,12].

Among toxins, alcohol and cocaine play a fundamental role; indeed, recent studies indicate that alcohol can be present in 21–36% of dilated cardiomyopathies [7,13]. Numerous drugs such as doxorubicin, anthracyclines, and trastuzumab can lead to dilated cardiomyopathy [14,15]. Then there are all the endocrinological causes, including takotsubo syndrome, which can lead to stress-related dilated cardiomyopathy [7].

#### 2.1.1. Causes

The main causes of idiopathic dilated cardiomyopathy are genetics. In this regard, Weintraub et al. highlights how 35% of dilated cardiomyopathies are linked to genetic alterations [7]. In particular, it has been reported that most of the mutations are autosomal dominant, less frequently X-linked, autosomal recessive, or mitochondrial patterns [6,7,16]. The genes most involved are:

○ TTN gene.

In particular, 25% of dilated cardiomyopathies are linked to mutations of the TTN gene present on chromosome 2 and encoded for the protein *titin*, which connects actin and myosin. The mutations that may occur are nonsense or frameshift mutations, splice variants, or insertions. These mutations result in a truncating variant of titin. An interesting element to consider is that the truncating variant of TTN is present in 1% of the healthy population. However, it has been reported that the presence of this variant is aggravated by excessive alcohol consumption and viral infections, and probably may also play a role in peripartum cardiomyopathy [17,18,19,20].

○ LMNA gene.

About 5% of dilated cardiomyopathies are due to mutation of the LMNA gene present on chromosome 1, which encodes for protein lamins A and C. These lamins assemble to create hetero-tetra-dimers that stabilize the internal nuclear lamina. The mutation of these proteins determines a distorted alpha helix and therefore the inability to form multimers to stabilize internal nuclear lamina. This mutation is also often associated with atrioventricular blocks, atrial fibrillation, and ventricular arrhythmias. In addition, mutations of the LMNA gene are also present in neuromuscular pathologies, such as muscular dystrophy Emery-Dreifuss (EDMD), limb-girdle-dystrophy, axonal neuropathy type 2 Charcot-Marie-Tooth, and familiar partial lipodystrophy. In particular, in these pathologies they can be linked to an X-linked or autosomal recessive inheritance [21,22].

○ Mutations in the Phospholamban (PLN) and Filamin C (FLNC) genes.

Both appear to be associated with 1–5% of dilated cardiomyopathies, and patients with FLNC mutations appear to have a more arrhythmogenic pattern such as those with LMNA mutations [6,16,23].

○ Other mutations that can cause dilated cardiomyopathies are those related to the following:

Genes of dystrophin (DMD), cardiac actin (ACTC), desmin (DES), the cardiac isoforms of beta-myosin-heavy chain (MYH7), troponin T (TNNT2), troponin I (TNNI3), delta-sarcoglycan (SGCD), sodium channel, type V (SCN5A), and desmoplakina (DSP) [12,16].

#### 2.1.2. Clinical Manifestation

Patients with dilated cardiomyopathy typically present with signs of congestive heart failure, such as dyspnea, congestive edema, orthopnea. The patients may also present arrhythmias (especially those in which mutations of the LMNA and FLNC genes are present), and sudden cardiac death may also occur [24].

#### 2.1.3. Diagnosis

##### Echocardiography

Echocardiography is often the test for the first diagnosis. Usually, ventricular dilatation with diffuse hypokinesia is found. This test can both address a possible cause, genetic, sarcoidosis, or infectious (myocarditis), and can also give prognostic information. About 40% of patients with dilated cardiomyopathy develop left ventricular reverse remodeling (LVRR), which has the worst prognosis. Typical LVRR changes, such as involvement of other chambers, increased size of the left atrium, functional mitral insufficiency, and alteration of diastole may be found with echocardiography [12,25].

##### EKG

The EKG is certainly an exam to be performed in the work up of dilated cardiomyopathy and to be carried out in the first evaluation. Ref. [26] can give some types of indications. There may be disorders compatible with ventricular hypertrophy, pathological Q waves or repolarization disorders, or conduction alterations such as prolongation of PR, typical of lamin alterations, emerine SCN5A, or even AV blocks, left bundle branch block (LBBB), and hemiblock [24,25,26]. The presence of atrial fibrillation, or LBBB, may be indicative of LVRR development, and therefore is associated with a prognostically unfavorable significance [25].

##### Laboratory Test

Laboratory tests can give information of an etiological nature. For example, an increase of CPK levels can suggest a dystrophin-related disorder, a laminopathy, or, more rarely, a disease of sarcoglycans, desminopathy, or a myofibrillar myopathy. An increase of TSH levels can suggest endocrinological causes. Other tests (such as HIV, Chagas, Borrelia) can suggest infectious diseases. An increase of thiamine levels can suggest alcohol abuse and finally an increase on BNP, renal function level can suggest a prognostic stratification [25,27].

##### Cardiac Magnetic Resonance Imaging (MRI)

MRI is also indicated in the initial work up for dilated cardiomyopathy. It can give information on the etiology. In particular, a late enhancement of gadolinium is present when there is necrosis or a scar that may indicate the presence of inflammation, especially if associated with characteristic images for edema and hyperemia [28,29].

##### Coronary Angiography

It is indicated in the diagnostic work up as it excludes a possible ischemic etiology [25].

##### Endomyocardial Biopsy

It is indicated only when a diagnosis that influences the therapy can be made, therefore in the suspicion of myocarditis, sarcoidosis, hemochromatosis [7,25].

##### Genetic Testing

The genetic basis of dilated cardiomyopathy requires genetic tests to be carried out on family members of patients with dilated cardiomyopathy. In fact, up to 50% of patient families can present pathogenic gene mutation. These are emerging approaches for more extensive genetic analysis [7,30]. Another important element is the genotype–phenotype relationship that can give fundamental prognostic information. For example, the carriers of LMNA mutations can indicate the development of ventricular arrhythmias that can be mortal and therefore with this knowledge they can be prevented [25].

#### 2.1.4. Management

The management of the patient with dilated cardiomyopathy involves managing and preventing the two pathologies with higher mortality linked to dilated cardiomyopathy: heart failure and arrhythmias [7]. The management of congestive heart disease involves the use of diuretics (furosemide) and vasodilators (nitrates) in patients with the *warm* and *wet* form, while it involves the use of inotropes in the *cold* and *wet* form [31]. Chronic heart failure must be managed with drugs normally used, such as ACE inhibitors and ARB (useful in the prevention of LVRR), beta blockers, angiotensin receptor neprilysin inhibitor (ARNI) (e.g., sacubitril-valsartan), mineralocorticoid antagonists, ivabradine, furosemide (useful in patients with congestive diseases, but does not greatly modify mortality), digoxin (in patients with heart failure and atrial fibrillation) [7].

Furthermore, genetic information can also be useful in therapy, as the forms in which there is a mutation of the SCN5A gene respond poorly to conventional therapy. Their phenotype can be managed with drugs that inhibit sodium channels, such as amiodarone and flecainide [7,32].

For the prevention of ventricular arrhythmias, the installation of electrical devices, such as implantable cardioverter defibrillators (ICD), is indicated in these patients [33]:

○ Patients who have survived a ventricular tachycardia, or;
○ Patients who have had symptomatic ventricular tachycardia, and;
○ In primary prevention in post-ischemic dilated cardiomyopathy.

Biventricular pacing is indicated in patients with symptomatic brady-arrhythmias [34]. The surgical approach involves cardiac transplantation, correction of mitral insufficiency or LVRRR and mechanical support (ECMO) [7].

An interesting element in the management of patients with dilated cardiomyopathy is the possibility of pharmacologically treating patients who have not yet developed symptoms, but who have compatible mutations (positive genotype-negative phenotype). To this regard, two clinical trials have shown that the early use of carvedilol or eplerenone may favorably impact the outcome [35,36]. Another very active field of study is the use of stem cells in patients with dilated cardiomyopathy and reduced ejection fraction, but there are still not enough studies on this [37].

### 2.2. Hypertrophic Cardiomyopathy (HCM)

It is the most common inherited cardiomyopathy due to mutations in numerous genes, encoding sarcomere proteins and is transmitted with an autosomal dominant pattern with variable penetrance. HCM is characterized by cardiac hypertrophy, particularly of the left ventricle (LV) (wall thickness ≥ 15 mm), in the absence of overload conditions (e.g., hypertension, valvular disease, etc.), which could justify this thickening [38,39]. In particular, in an adult, HCM is defined by a wall thickness >15 mm in one or more MV myocardial segments, as measured by an imaging technique, and is not explained solely by loading conditions. As in adults, in children, the diagnosis of HCM requires wall LV thickness more than two standard deviations greater than the predicted mean [38]. In the literature, it was first described in 1868 by Vulpian et al., who defined it as idiopathic hypertrophic subaortic stenosis [40]. This and the subsequent descriptions by Broke and Teare, in the 1950s, devoted much attention to the obstruction of the outflow tract of the left ventricle (LVOTO). These data in fact are present in about 70–75% of patients with HCM and this constitutes hypertrophic obstructive cardiomyopathy [41,42,43]. Clinically, HCM can remain asymptomatic or paucisymptomatic for a long time. Some of the symptoms include exertional dyspnea, chest pain, syncope, and palpitations. These symptoms can be associated with ventricular and supraventricular arrhythmias. In some cases, fortunately rare, the first clinical manifestation is sudden cardiac death (SCD). The prevalence of HCM reported is equal to 1:500 (0.2%) [44,45,46]. The prevalence of HCM has been underestimated for years because the echocardiogram has a lower sensitivity than magnetic resonance imaging (MRI) [47,48]. Some author defines, considering some correction factors, the estimated prevalence as at least 1:200 (0.5%) [49]. Women are older at diagnosis than men, have greater symptom severity (NYHA class), and are more likely to have left ventricular outflow tract [50,51].

#### 2.2.1. Causes

In about 40% of HCM patients, the causal genes remain unidentified. The pathophysiological mechanisms by which sarcomeric gene mutations give HCM are not yet fully understood [52].

The genes most involved are:

○ MYBPC3 gene (locus 11p11.2). This encodes cardiac myosin-binding protein C of the intermediate filament. Several mutations of this gene have been identified as missense, nonsense, splicing, deletion, and insertion. It is the most common gene involved, representing up to 40% of mutations [53].
○ MYH7 gene (locus 14q11.2). This encodes beta-myosin heavy chain of thick filament. It is present in about 15–25% of patients with HCM [53,54,55].
○ TNNT2 gene (locus 1q32.1). This encodes cardiac muscle troponin T of thin filament. It represents 5–10% of cases [55].
○ TNNI3 gene (locus 19q13.4). This encodes cardiac troponin I of thin filament and is present in 4–8% of cases [56].
○ Rare genes involved are:
○ MYL2 gene (locus 12q23-q24) that encodes regulatory myosin light chain of thick filament [57].
○ MYL3 gene (locus 3p21.3) that encodes essential myosin light chain of thick filament [57].
○ TPM1 gene (locus 15q22.1) that encodes α-tropomyosin of thin filament [58].
○ ACTC1 gene (locus 15q11q14) that encodes α-cardiac actin of thin filament [59].

#### 2.2.2. Clinical Manifestations

**Figure 2:**
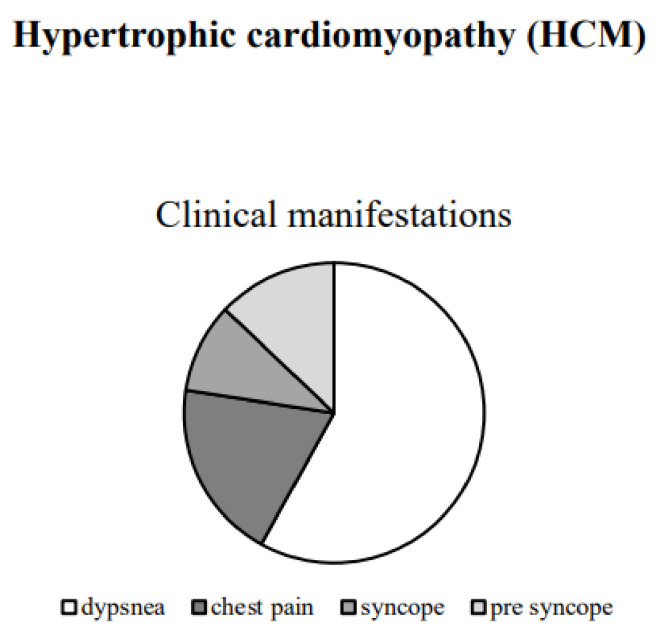
In this figure, we report the clinical manifestation in HCM (%).

Many patients with HCM have no or only minor symptoms throughout life. Dyspnea on exertion, as a symptom of heart failure (HF), is present in more than 90% of symptomatic patients [60]. Typical chest pain on exertion occurs in 25 to 30% of patients with HCM [61]. Syncopal episodes occur in about 15–25% of patients with HCM. Another 20% of these patients report pre-syncope episodes [62]. HCM can present with both supraventricular and ventricular arrhythmias. These can appear to the patients as palpitations, dyspnea, presyncope/syncope (Figure 2).

The most common supraventricular is, as in the general population, atrial fibrillation. Among the ventricular arrhythmias, episodes of non-sustained ventricular tachycardia (NSVT), are present in about 75% of patients [63,64]. About 8% of patients, unlike the vast majority, present an evolution of the pathology that leads to a remodeling of the left ventricle with a reduction of the ejection fraction of the left ventricle <50%. They generally have more severe HF symptoms, and have a higher risk of sudden cardiac death (SCD) [65]. Two murmurs are common in HCM [53]:

○ A systolic murmur that begins slightly after S1 and is heard best at the apex and lower left sternal border, due to LVOTO obstruction.
○ A holosystolic murmur heard loudest at the apex which radiates to the axilla, due to mitral regurgitation.

#### 2.2.3. Diagnosis

The diagnosis of HCM, being a hereditary disease, inevitably starts from the medical history. In particular, the family history can help to identify family members diagnosed with HCM. Another important aspect is the recognition of “red flags” that guide the rational selection of further diagnostic tests as reported by [27]. The electrocardiogram is also useful, as it may present anomalies. The echocardiogram (EKG) and/or cardiac MRI are obviously aimed at identifying structural anomalies. In particular, they can help to identify the thickness of the left ventricular wall that, when it is >15 mm, constitutes a diagnosis, in the absence of overload conditions (e.g., hypertension, valvular disease, etc.) [43].

##### EKG

The electrocardiogram is the most sensitive test for HCM, with abnormal results in more than 90% of cases [66]. For this reason and the low cost of execution it is usually the first test performed in the diagnostic process. However, the low specificity of this test should be considered, so that no anomaly found will be pathognomonic. The most common electrocardiographic findings are: P wave anomalies (left or bilateral atrial dilation index), prominent Q waves in the lateral (I, aVL, V4–V6) and lower (II, III, aVF) leads, repolarization anomalies, left axial deviation. Deep and inverted T waves can instead be found in the precordial ones (V2–V4) and are suggestive of the mid-ventricular or apical variant of HCM [56].

##### Dynamic EKG Holter

It is not useful for diagnosis, but it is essential for the identification of arrhythmias. Non-sustained ventricular tachycardia (NSVT), in particular, correlates with sudden cardiac death (SCD). Although routine monitoring lasts 24–48 h, recent studies have shown that continuous monitoring for up to 14 days has a significantly higher sensitivity. The high prevalence of NSVT in these patients seems to question its usefulness for risk stratification of SCD [64].

##### Echocardiography

Transthoracic echocardiography (TTE) should be performed in every patient with a suspected diagnosis of HCM. Only the wall thickness >15 mm of the left ventricle is indispensable for the diagnosis of HCM. Other findings are:

○ Diastolic dysfunction;
○ Enlargement of left atrium that is associated with increased risk of atrial fibrillation;
○ Systolic dysfunction evaluated with global longitudinal strain (GLS), which is associated with major risk of heart failure, even with a normal LV ejection fraction [67].
○ LVOTO obstruction due to systolic anterior motion (SAM) of the mitral valve. The obstruction is defined as a maximal left ventricular gradient >30 mmHg at rest or during exercise or provocative manoeuvers (such as Valsalva) [56].
○ Exercise stress testing;

Until a few decades ago, the stress test was underutilized as HCM was considered a relative contraindication. However, several subsequent studies have shown that this test is safe and essential in the diagnostic-therapeutic process of HCM. It is useful to identify outflow tract obstruction in patients with no gradient at rest, abnormal blood pressure response, coexistent coronary artery disease, provocable ventricular arrhythmias, and consequently it evaluates the risk stratification for SCD [68,69,70].

##### Cardiovascular Magnetic Resonance (CMR)

CMR should be performed in all the patients with HCM. It allows one to collect more information on cardiac anatomy compared to transthoracic echocardiography (TTE) [71,72]. It shows in detail the septum, the mitral valve, and the papillary muscles, useful for the preoperative evaluation before ventricular septal myectomy [73]. With the injection of contrast, as late gadolinium enhancement (LGE), CMR detects typical patterns of hyper-enhancement that can differentiate HCM from other so-called non-sarcomeric causes that could mimic HCM, such as Anderson–Fabry disease [67]. Furthermore, myocardial fibrosis, detected with LGE, appears to increase the risk of ventricular arrhythmias and SCD in patients with HMC [74,75]. CMR also shows in detail the septum, the mitral valve, and the papillary muscles, useful for the preoperative evaluation before ventricular septal myectomy [73].

##### Genetic Test

Genetic testing to detect the presence of specific pathogenic mutations is not available worldwide. Not all mutations underlying HCM are known yet. Furthermore, because of the variable penetrance of these mutations, to have the mutation does not mean to be affected by HCM.

Patients with the pathogenic mutation, but in the absence of disease, are so-called “genotype positive/phenotype negative” patients. These patients could manifest the disease during their life. For this reason, they are eligible for periodic follow-up with EKG, echocardiography, CMR etc. On the other hand, these tests are mainly used in first degree relatives of patients who already have a known pathogenic mutation, to identify those who have a higher risk of developing HCM. Relatives with no mutation can be discharged from the follow-up [39,76]. Another important use of the genetic test is to differentiate HCM from other syndromes, so-called non-sarcomeric causes, due to the presence of left ventricle hypertrophy, which may require different management. For example, Fabry disease is caused by mutations in the gene encoding alpha-galactosidase A [77]; in Noonan syndrome, the left ventricular hypertrophy (LVH) is due to mutations in the genes coding for components of the RAS MAPK pathway [78]; in the glycogen storage cardiomyopathy, LVH is associated with mutations in genes encoding a subunit of adenosine monophosphate (AMP)-activated protein kinase (PRKAG2) and lysosome-associated membrane protein 2 (LAMP2) [79].

#### 2.2.4. Management

For asymptomatic patients, a conservative approach is preferred. No medication is indicated but periodic follow-up to evaluate the disease evolution is fundamental (39). Left ventricular outflow tract (LVOTO) is defined as rest or provocated LV outflow tract gradient >30 mmHg. In patients with HCM, the symptoms of progressive HF (fatigue, dyspnea) or chest pain in 90% of cases are due to LVOTO. These can be treated first with beta-blockers. Alternatively, if beta-blockers are ineffective or not tolerated, disopyramide when available can be used, or calcium-channel blockers (verapamil and diltiazem). Weight loss must be encouraged. Hypovolemia should be avoided and for this reason vasodilators and diuretics are not indicated [43]. If pharmacological therapy is ineffective or LV outflow gradient is >50 mmHg, invasive treatment can be performed. In this case, usually the first choice to treat LVOTO is ventricular septal myectomy (Morrow procedure) [80]. Second choice is represented by septal alcohol ablation [81].

About 10% of patients with HCM and heart failure symptoms have no LVOTO. In these patients, symptoms are probably sustained by diastolic dysfunction and reduced ventricular filling, even though these alterations could be undetectable with echocardiography [82]. In these patients with no obstruction, if LVEF is >50%, to treat the symptoms, the drugs indicated are ß-blockers, verapamil or diltiazem, low dose loop and thiazide diuretics. If LVEF < + 50% ß-blockers, ACE-i, mineralocorticoid receptor antagonist (MRA), low dose loop and thiazide diuretics can be used [38].

Atrial fibrillation is the most common supraventricular arrhythmia in patients with HCM. It can be responsible for symptoms such as palpitations or dyspnea, and it could worsen the symptoms of heart failure. The treatment is comparable to that used in the general population, but it should be emphasized that patients with HCM are less tolerant to high heart rates and they have a greater thromboembolic risk. In this regard, a more aggressive approach to rhythm control is needed and the introduction of prophylactic anticoagulant therapy without delay is preferable [83].

NSVT is a common finding in routine EKG and is a risk factor for SCD but usually no anti-arrhythmic therapy is required. No evidence exists that sustained monomorphic ventricular tachycardia (VT) that is well tolerated has a worse prognosis compared to NSVT. Patients that do not tolerate VT could be eligible for implantable cardioverter defibrillators (ICDs) therapy and treatment with ß-blockers or amiodarone for secondary prevention (38).

Treatment with mavacamten improved exercise capacity, LVOT obstruction, NYHA functional class, and health status in patients with obstructive hypertrophic cardiomyopathy. The results of this pivotal trial highlight the benefits of disease-specific treatment for this condition. Mavacamten is effective for treatment of symptomatic obstructive hypertrophic cardiomyopathy [84].

### 2.3. Sudden Cardiac Death (SCD)

It has been reported that HCM is the most important cause of sudden death on the athletic field in the United States [72]. In preventive strategies for SCD, competitive sport and strenuous exercise should be discouraged in these patients. Implantable cardioverter defibrillators (ICDs) are recommended for secondary prevention for patients with a history of cardiac arrest due to VT or ventricular fibrillation (VF) or spontaneous sustained VT causing syncope or hemodynamic compromise (38). In primary prophylaxis, the decision to implant an ICD is done case by case. It is usually implanted when at least one of the following major risk markers [43] is present:

○ Family history of HCM-related sudden death;
○ Massive LVH (≥30 mm);
○ Unexplained syncope;
○ End stage HF (ejection fraction <50%);
○ Multiple, repetitive NSVT;
○ Extensive LGE;
○ LV apical aneurysm.

If the level of risk remains uncertain, to help make the decision potential risk mediators are considered:

○ Marked LV outflow obstruction at rest;
○ Hypotensive response to exercise;
○ Age ≥ 60 years (reduced risk);
○ Alcohol septal ablation.

### 2.4. Arrhythmogenic Cardiomyopathy (ARCV)

Arrhythmogenic cardiomyopathy (ARCV) is an arrhythmogenic disorder of the myocardium not secondary to ischemic, hypertensive, or valvular heart disease. ARCV incorporates a broad spectrum of genetic, systemic, infectious, and inflammatory disorders. This designation includes, but is not limited to, arrhythmogenic right/left ventricular cardiomyopathy, cardiac amyloidosis, sarcoidosis, Chagas disease, and left ventricular noncompaction. The ARCV phenotype overlaps with other cardiomyopathies, particularly dilated cardiomyopathy with arrhythmia presentation that may be associated with ventricular dilatation and/or impaired systolic function [85]. In 1968 in France, there was the first demonstration of an infiltration of fibrofatty tissue in the right ventricle [86]. Initially it was called arrhythmogenic right ventricular dysplasia; then it became arrhythmogenic right ventricular cardiomyopathy. The term “arrhythmogenic cardiomyopathy” is used to describe a family of diseases that features structural myocardial abnormalities (identified by macro and microscopic pathological examination besides cardiac imaging) and ventricular arrhythmia [87]. The manifestations were more present in the right ventricle, but over time it was understood that the left ventricle could also be involved equally to the right or it could be predominant [88,89,90]. The prevalence of arrhythmogenic cardiomyopathy ranges from 1:1000 to 1:5000. This variability is linked to the fact that sudden cardiac death is often the presentation and arrhythmogenic cardiomyopathy is not recognized as a cause in 30% of cases [91,92]. A characteristic element is the high prevalence in Northeast Italy [93]. There, cardiomyopathy has the highest prevalence in cases of sudden cardiac death. In fact, European and American studies indicate that, in the post mortem evaluations of subjects with sudden cardiac death, arrhythmogenic cardiomyopathy was present in 20–31% [93].

#### 2.4.1. Causes

Arrhythmogenic cardiomyopathy is a disease with a genetic basis, and it is characterized by the progressive replacement of the myocardium with fibrofatty tissue that progressively starts from the epicardium to become transmural, with the development of multiple aneurysms. The localization typically is in the dysplasia triangle, which includes apex, influx tract, and outflow tract of the right ventricle, but often also involves the left ventricle (up to 76% of cases) [94,95]. Another interesting element is the finding of a viral genome in autopsies, suggesting an infectious cause. Most likely viruses are not the cause and myocardial degeneration could encourage a viral infection [96]. From the genetic point of view, the most important mutations related to arrhythmogenic cardiomyopathy are those of the desmosome genes.

The most involved genes are:

○ JUP,
○ DSP,
○ PKP2,
○ DSG2,
○ DSC2.

The JUP mutation causes Naxos disease, which is a disease typical of the Greek island in which patients have palmoplantar keratoderma, woolly hair, and arrhythmogenic cardiomyopathy with a recessive pattern. The mutations of the DSP gene have been found in South America in a recessive disorder characterized by keratoderma, wooly hair, and arrhythmogenic cardiomyopathy, but with a prevalence of the left ventricle.

Other genes involved are those linked to the nuclear envelope:

○ LMNA and TMEM43 genes.

Then there are mutations of the composite area. The area composita is a mixed type of junctional structure composed of both desmosomal and adherens junctional proteins.

However, there are genes in common with other cardiomyopathies (such as DES, PLN, TGFB3, TTN, SCN5A) [95].

#### 2.4.2. Clinical Manifestation

Arrhythmogenic cardiomyopathy can vary its phenotypic expression, from completely asymptomatic subjects to the development of ventricular arrhythmias that can even be fatal and lead to sudden cardiac death [97]. The most typical clinical presentation is that related to arrhythmias that can cause palpitations, syncope (often during exercise), up to cardiac arrest [93]. However, it has been described as a clinical presentation that can also simulate myocarditis with changes in EKG repolarization and chest pain [98]. There are four natural stages for the ARCV:

○ The concealed phase, in which there are no or subtle structural changes in the right ventricle, with or without minor ventricular arrhythmias. In this case, sudden cardiac death may occur even at this early stage as the first manifestation of the disease in previously asymptomatic young individuals.
○ The second phase is characterized by the occurrence of arrhythmias in association with manifest functional and structural abnormalities in the right ventricle, which are detectable by current imaging tests. Patients may experience arrhythmic symptoms such as palpitations, syncope, or cardiac arrest.
○ The third phase is characterized by right ventricular (RV) failure with a relatively preserved LV function.
○ The end stage is characterized by parallel significant left ventricular (LV) involvement with systolic dysfunction. At this stage, AC can mimic dilated cardiomyopathy of other causes with its related complications, such as atrial fibrillation and thromboembolic events.

Sudden cardiac death is typically due to the development of fatal ventricular arrhythmias, due to the development of reentry circuits in the areas where fibrofatty scar develops. Another very interesting hypothesis proposes a cross-talk between desmosomes and sodium channels which can lead to the development of fatal arrhythmias. This hypothesis can be supported by the finding that even in the first phase of the disease, where there are no structural changes in ventricles, the patients can have arrhythmias and sudden cardiac death [99,100].

#### 2.4.3. Diagnosis

In 1994, an international task force formulated diagnostic criteria for arrhythmogenic cardiomyopathy of the right ventricle, and then modified them in 2010. The criteria include diagnostic test, histological/biopsy, and anamnestic evaluations.

##### Echocardiography

It has been observed that global or segmental changes in the kinetics and structural changes of the ventricle can also be evaluated with MRI or with catheterization of the right ventricle.

##### EKG

The most characteristic diagnostic element is the presence of inverted T waves in the anterior leads, with a prevalence ranging from 19% to 94%, the presence of an Epsilon wave (reproducible low-amplitude signals between the end of the QRS complex to the onset of the T wave), presence of right branch bundle block, fragmented QRS, and the finding of ventricular arrhythmias.

##### Biopsy

The most characteristic diagnostic element is less than 60% of residual myocardial cells with fibrous replacement on more than one sample taken from the free wall of the right ventricle.

##### Family History

Presence of confirmed ablation in arrhythmogenic right ventricular cardiomyopathy (ARVC) in a first-degree family member or premature death in a first-degree family member is very suggestive for AC [89,101].

#### 2.4.4. Management

The goal in the management of arrhythmogenic cardiomyopathy is to prevent sudden cardiac death. The first step is to impose lifestyle change. In fact, it has been observed that during exercise the risk of developing ventricular arrhythmias increases. Therefore, patients with arrhythmogenic cardiomyopathy should be prohibited from competitive or endurance sport activity [102,103]. The pharmacological approach instead involves the use of beta-blockers that reduce adrenergic activity and therefore the risk of developing arrhythmias. Other drugs used in patients with positive phenotype, are amiodarone and sotalol together with beta-blockers, especially in patients with premature ventricular beats or with non-sustained ventricular tachycardia [103,104]. In patients with monomorphic ventricular tachycardia, there is an indication for catheter ablation [105].

The ICD implantation according to the task force is indicated in the following cases:

○ The high-risk category (estimated event rate >10% per year) includes either patients with a history of cardiac arrest or sustained VT or patients with severe dysfunction of the RV, LV, or both. The indication for ICD implantation in this subset of patients is a class I recommendation.
○ For the intermediate-risk category (estimated event rate of 1–10% per year), which includes patients with ≥1 risk factor and no previous malignant arrhythmic events, the indications for ICD therapy for primary prevention of sudden cardiac death (SCD) are the following:
○ In the presence of major risk factors such as syncope, non-sustained VT, or moderate ventricular dysfunction, an ICD can be recommended (class IIa).
○ In selected patients with ≥1 minor risk factor, where the arrhythmic risk is not sufficiently high or defined, ICD therapy may also be considered (class IIb).
○ Prophylactic ICD implantation is not recommended (class III) in asymptomatic patients with no risk factors and in healthy gene carriers (low-risk category), event rate <10% per year [103].

Finally, new therapeutic approaches are present above all in the field of translational medicine. In fact, medicines that activate Wnt signaling, blocking GSK3β, seem to be useful in animal models. In man, there are still many perplexities; moreover, PPARγ and PPARα seem to be possible targets for the treatment of arrhythmogenic cardiomyopathy and finally stem cells are also the subject of current debate [106,107,108,109].

### 2.5. Restrictive Cardiomyopathy (RCM)

Restrictive cardiomyopathy (RCM) is a heart-muscle disease characterized by stiffness of the ventricular walls leading to diastolic dysfunction, raised end-diastolic pressure, and dilated atria. The ventricles are not dilated and there is physiological wall thickness. Therefore, systolic function is usually preserved. Impairment of the ventricular structure and its systolic function may be present only in the advanced stages of secondary RCM [110]. RCM is not a single disease but can be the result of multiple inherited or acquired predispositions. As in other cardiomyopathies, also in RCM there are genetic mutations in the genes encoding the sarcomere proteins that have been associated. Epidemiology of this disease in not so well represented in literature, but RCM is the least common of the cardiomyopathies. An idiopathic pattern in which no identifiable cause is found is a really rare disease. It can affect people at any age. Children have the worst prognosis and girls seem to be more affected [111]. It can be acquired or inherited. In the latter case, for each cause some peculiar gene mutations have been identified.

#### 2.5.1. Causes

RCM is classified in:

○ Infiltrative:
  (a) Amyloidosis (acquired/inherited);
  (b) Genes: TTR gene variants (V122I; I68L; L111M; T60A; S23N; P24S; W41L; V30M; V20I), APOA1;
  (c) Sarcoidosis (acquired);
  (d) Primary hyperoxaluria (inherited).

○ Storage disease:
  (a) Fabry disease (inherited). Gene: GLA;
  (b) Gaucher disease (inherited). Gene: GBA;
  (c) Hereditary hemochromatosis (inherited). Genes: HAMP, HFE, HFE2, HJV, PNPLA3, SLC40A1, TfR2;
  (d) Glycogen storage disease (inherited);
  (e) Mucopolysaccharidosis type I (Hurler syndrome) (inherited). Gene: IDUA;
  (f) Mucopolysaccharidosis type II (Hunter syndrome) (inherited). Gene: IDS;
  (g) Niemann–Pick disease (inherited). Genes: NPC1, NPC2, SMPD1.

○ Non-infiltrative:
  (a) Idiopathic (acquired);
  (b) Diabetic cardiomyopathy (acquired);
  (c) Scleroderma (acquired);
  (d) Myofibrillar myopathies (inherited). Genes: BAG3, CRYAB, DES, DNAJB6, FHL1, FLNC, LDB3, MYOT;
  (e) Pseudoxanthoma elasticum (inherited). Gene: ABCC6;
  (f) Sarcomeric protein disorders (inherited). Genes: ACTC, β-MHC, TNNT2, TNNI3, TNNC1, DES, MYH, MYL3, CRYAB;
  (g) Werner’s syndrome (inherited). Gene: WRN.

○ Endomyocardial:
  (a) Carcinoid heart disease (acquired);
  (b) Endomyocardial fibrosis idiopathic (acquired);
  (c) Hypereosinophilic syndrome (acquired);
  (d) Chronic eosinophilic leukemia (acquired);
  (e) Drugs (serotonin, methysergide, ergotamine, mercurial agents, busulfan) (acquired);
  (f) Endocardial fibroelastosis (inherited). Genes: BMP5, BMP7, TAZ;
  (g) Consequence of cancer/cancer therapy: metastatic cancer, drugs (anthracyclines), radiation (acquired).

#### 2.5.2. Clinical Presentation

The clinical presentation obviously depends on the associated pathology in the case of secondary RCMs. However, in all RCMs and, in particular, in the idiopathic pattern, diastolic impairment can affect both the left and right ventricles. The signs and symptoms of RCM mirror both systemic and pulmonary congestion. More common symptoms are dyspnea and pulmonary edema, but even palpitations, fatigue, orthopnea, and chest pain can be present. On clinical examination, the following can be found: mostly jugular venous distension, systolic murmur, third heart sound, pulmonary rales, and peripheral edema. Hepatosplenomegaly, ascites, and anasarca are rarer and only present in advanced stages of the disease [110]. Some recent studies show how, in patients with RCM, cardiac and peripheral autonomic dysfunction is associated with reduced baroreflex sensitivity, causing clinical deterioration and arrhythmias. The presence of a normal ejection fraction underestimates the evolution of the disease [111,112]. Clinically, RCM is not distinguishable from constrictive pericarditis (CP). In particular, history of cardiac surgery, trauma, tuberculosis, malignancy, etc. are more suggestive of constrictive pericarditis, while high plasma levels of BNP are more suggestive of restrictive cardiomyopathy than CP [113]. On chest radiography, the finding of pericardial calcifications and/or low QRS voltages on EKG direct the diagnosis to CP. Tissue tracking-derived left ventricular mechanics on echocardiography and cardiac magnetic resonance (CMR) can provide further information to facilitate this distinction [114].

#### 2.5.3. Diagnosis

##### EKG

About 99% of patients with RCM have EKG abnormalities. Atrial fibrillation is most common in arrhythmia, even because of the atrial enlargement. Elevated ST segment with notched or biphasic late peaking T waves is a common finding. Moreover, significant ST depression with T inversion mimicking subendocardial ischemia is described in some cases of RCM and it seems to be associated with an increased risk of SCD. Premature ventricular and atrial beats can also be present [110,115].

##### Chest Radiography

The most common finding on chest radiography is cardiomegaly, due to bilateral atrial enlargement. Other findings can be pulmonary venous congestion, interstitial edema, and pleural effusion [110].

##### Echocardiography

Typically, echocardiography shows absence of ventricular hypertrophy or dilatation, preserved systolic LV ejection fraction, bilateral atrial enlargement, and diastolic dysfunction. The American Society of Echocardiography (ASE) suggests four parameters to identify diastolic dysfunction:

○ Atrial left (LA) maximum volume index >34 mL/m;
○ Tricuspid regurgitation peak velocity (TRV) >2.8 m/s;
○ Average E/e’ ratio >14;
○ Annular e’ velocity (septal e’ <7 cm/s, lateral e’ <10 cm/s).

The ratio of pulmonary vein peak systolic to peak diastolic velocity, and the changes in E/A ratio with Valsalva manoeuver, are used to show the increasing of LV filling pressures. Some echocardiographic findings also play a key role to help the differentiation between an apparent idiopathic form of RCM from secondary ones (as in sarcoid heart disease, hypereosinophilic syndrome, diabetic cardiomyopathy, scleroderma, endomyocardial fibrosis, radiation, carcinoid heart disease, metastatic cancers, etc.). Furthermore, they provide additional data to distinguish RCM from constrictive pericarditis [116,117,118].

##### Cardiac Magnetic Resonance (CMR)

CMR provides more information than echocardiography. In particular, it is useful to identify specific patterns characteristic of diseases causing RCM. For example, diffuse subendocardial late gadolinium enhancement (LGE) has a specificity of about 95% for the diagnosis of cardiac amyloidosis (CA). On the other hand, LGE on CMR has been identified as a valuable prognostic factor in patients with cardiac sarcoidosis or CA. Moreover, as echocardiography, CMR can be used to differentiate RCM from constrictive pericarditis [119,120].

##### Endomyocardial Biopsy

Endomyocardial biopsy is principally performed when other tests are inconclusive. It can detect, for example, the presence of amyloid or iron deposition to confirm or exclude some secondary RCMs. Nevertheless, because of the patchy pattern of parts of this disease, it could be useful to perform LGE-CMR to guide this test and to decrease the likelihood of a false negative [110].

#### 2.5.4. Treatment

Secondary RCMs can have different management because it could depend on the particular genesis of the underlying disease. For the idiopathic form, the management is aimed at limiting the symptoms of HF [121]. To improve the ventricular filling and reduce the diastolic dysfunction, reducing the heart rate, non-dihydropyridine calcium channel blockers (e.g., verapamil, diltiazem) can be used. For the same reason, beta-blockers can be administrated. Moreover, this kind of drug has also shown a positive effect on ventricular relaxation. Arrhythmias should be treated and sinus rhythm should be preferred. If sinus rhythm is not restorable, oral anticoagulation has to be administrated. Implantable defibrillators for the risk of SCD are considered even if precise selection criteria can be difficult to define [122]. Heart transplantation is the only definitive treatment for RCM. It is reserved for patients with untreatable HF. By the way, some groups of study, because of the progressive course of this disease, recommend listing them for transplantation at the diagnosis, even in asymptomatic patients [123].

Loop diuretics are given to relieve venous congestion in the pulmonary and systemic circulation. High doses have to be avoided because an excessive drop of preload could reduce the cardiac output too much, causing hypotension. Digoxin should be used with caution because of its arrhythmogenic effect.

### 2.6. Takotsubo Cardiomyopathy

Takotsubo cardiomyopathy, also known as stress-induced cardiomyopathy or broken-heart syndrome, is defined as an abrupt onset of left ventricular dysfunction in response to severe emotional or physiologic stress (1). Post-menopausal women are most commonly affected. The exact prevalence has been estimated at 0.02% of hospitalized patients. It has been reported that takotsubo cardiomyopathy accounts for 1–2% of admissions for acute coronary syndrome [124,125]. It often presents with angina. Typical ischemic changes may be seen with EKG and with elevated cardiac enzymes [124]. On echocardiography, a pattern of apical ballooning of the left ventricle has been reported. Because its presentation closely mirrors that of acute coronary syndrome, takotsubo cardiomyopathy initially should be treated in the same way. Acute complications, such as shock or heart failure, should be managed appropriately. Stable patients may be treated with diuretics, ACE inhibitors or ARBs, and beta-blockers [124]. Anticoagulants should be provided to patients with loss of wall motion in the left ventricular apex [124]. Symptoms and abnormalities typically reverse within one month, and treatments may be withdrawn accordingly [124,125].

### 2.7. Peripartum Cardiomyopathy

Peripartum cardiomyopathy is a cause of heart failure during pregnancy and the peripartum period [126]. The ESC Working Group defined the following characteristics to identify peripartum cardiomyopathy:

○ Development of heart failure (HF) toward the end of pregnancy or within five months following delivery.
○ Absence of another identifiable cause for the HF.
○ Left ventricular (LV) systolic dysfunction with an LV ejection fraction (LVEF) of less than 45 percent. The LV may or may not be dilated [126].

The incidence is highly variable, ranging from 1:968 to 1:4000 live births in the USA, 1:10,149 in Denmark, 1:5719 in Sweden [127,128,129]. The etiology remains quite uncertain; some mechanisms have been considered, such as angiogenic imbalance. In this regard, studies have shown how the lack of the PGC-1α gene, a regulator of pro-angiogenic factors such as VEGF, can lead to the development of peripartum cardiomyopathy [130]. Some studies show how mice knockout in the cardiac tissue-specific signal transduction and activator of transcription 3 (STAT3) develop peripartum cardiomyopathy. Reduction in STAT3 leads to increased cleavage of prolactin into an antiangiogenic and proapoptotic 16kDa isoform by cathepsin D. This alteration in prolactin processing may contribute to the angiogenic imbalance [131]. The 16 kDa prolactin fragment also causes endothelial damage and myocardial dysfunction [132]. Other studies reported that there is an increased presence of TNF alpha and Il-6 in women with peripartum cardiomyopathy, so there may be a correlation between cytokines and peripartum cardiomyopathy [133]. Some authors have proposed a genetic predisposition, evaluating the frequent overlap between peripartum cardiomyopathy and dilated cardiomyopathy on the basis of alteration of some genes such as that of titin [20]. Possible risk factors are [134,135,136,137,138]:

○ Age greater than 30 years;
○ African descent;
○ Pregnancy with multiple fetus;
○ Preeclampsia, eclampsia;
○ Cocaine abuse;
○ Long-term use (>4 weeks) of tocolytics (terbutaline).

The most typically observed symptoms are dyspnea, cough, orthopnea, nocturnal dyspnea, peripheral edema, fatigue; these latter symptoms are not very specific during pregnancy. Possible complications can be arrhythmias and the development of thromboembolism [139,140].

The diagnosis is mostly clinical, but EKG, laboratory, and radiological tests may present alterations:

○ EKG: in 50%, it presents anomalies such as sinus tachycardia, repolarization anomalies, Q waves [141];
○ BNP: BNP is typically high [142];
○ Chest X-ray: Enlargement of the cardiac silhouette, redistribution of flow, and pleural effusion may be found [143];
○ Echocardiography: Reduction in left ventricular ejection fraction (<45%) and frequent left ventricle dilatation [144].

There are few studies about novel markers, such as plasma concentrations of proangiogenic and antiangiogenic factors, including placenta growth factor, fms-like-tyrosine-kinase 1 receptor, and their ratios, which have been proposed to be used to distinguish patients with peripartum cardiomyopathy, but other studies are needed [145]. The management of peripartum cardiomyopathy therefore follows the guidelines of the management of heart failure: adequate oxygen must be administered, preload optimized, inotropes administered, if necessary, for relief of symptoms. Arrhythmias must be managed and if necessary, an ICD must be implanted. Anticoagulant therapy [146] must be set up. There are currently experimental protocols under study: bromocriptine, intravenous immune globulin, antisense therapy against micronRNA-146a and apheresis [146,147,148,149]. As for the delivery, the decision must be shared in a team with the presence of the cardiologist, gynecologist, anesthetist, and neonatologist [146]. A hemodynamically stable patient can undergo vaginal delivery with epidural. In a woman with hemodynamic instability, an emergency delivery is necessary. In women with advanced heart failure and use of inotropes, a caesarean delivery should be planned [126]. As regards breastfeeding, there are no reliable data; some authors suggest that women with advanced heart failure should not breastfeed due to the potential role of prolactin, but certainly those who are hemodynamically stable should be encouraged to breastfeed [133,150]. The mortality of peripartum cardiomyopathy is 10% in two years, 6% in five years (11). Complete recovery of left ventricular function is reported in 20–70% of patients, with recovery usually within six months of diagnosis [151,152,153].

### 2.8. Cardiotoxicity and Chemotherapy Drugs

Cancer patients undergoing chemotherapy may develop cardiomyopathies. The agents most involved are anthracyclines and trastuzumab. The mechanism by which anthracyclines create myocardial damage may be linked to the development of oxygen free radicals (ROS) which increase oxidative stress and therefore create myocardial damage. More recent studies find the implication of the enzyme topoisomerase II; doxorubicin binds topoisomerase 2 and DNA forming a ternary complex leading to cell death, cardiomyocytes present topoisomerase 2 alpha and beta, and it appears that doxorubicin can bind cardiac topoisomerases, resulting in the death of myocytes [152,153]. Among the most implicated risk factors are [154,155]:

○ Old age (<65 years) or young (>4 years);
○ Female gender;
○ Pre-existing heart disease;
○ Hypertension;
○ Smoke;
○ Hyperlipidemia;
○ Obesity;
○ Diabetes;
○ High cumulative anthracycline exposure.

As for trastuzumab, a monoclonal antibody that targets the human epidermal growth factor receptor 2, the modality with which it determines cardiotoxicity is different from anthracyclines, because it does not cause myocardial damage, but an alteration to the contractility, which therefore makes the latter cardiomyopathy more frequently reversible and less linked to drug accumulation [156,157].

Risk factors for developing trastuzumab cardiomyopathy are [158,159]:

○ Over 50 years of age;
○ Previous or concomitant use of anthracyclines;
○ Obesity;
○ Preexisting cardiac dysfunction;
○ Hypertension.

The clinical manifestations of anthracycline cardiomyopathy are linked to early symptoms such as EKG abnormalities, arrhythmias, atrioventricular blocks, and pericarditis-myocarditis; vice versa, we can find late signs that are related to the development of heart failure such as dyspnea, asthenia, edema, orthopnea [159,160,161,162,163,164]. Other chemotherapy agents that can cause cardiomyopathies are:

○ Paclitaxel: Associated with doxorubicin, it has been shown to cause heart failure in 20% of patients [165,166];
○ Cyclophosphamide: Heart failure is found in patients with high dose protocols; negative prognostic factors are lymphoma, preceding mediastinal irradiation, advanced age, cardiac abnormalities [167,168];
○ Cisplatin: Cardiotoxicity due to cisplatin can be manifested by supraventricular tachycardia, bradycardia, ST-T wave changes, left bundle branch block, acute ischemic events, myocardial infarction, and ischemic cardiomyopathy. This toxicity may be related to electrolyte abnormalities secondary to cisplatin-induced nephrotoxicity [169,170].

## 3. Conclusions

In particular, it has been reported that HCM is the most important cause of sudden death on the athletic field in the United States [72]. It is needless to say how important it is to know which changes in the heart due to physical activity are normal, and when they are pathological. However, it is very crucial to achieve as a goal reaching the period in which pharmacological and invasive procedures will no longer be the only measures to manage cardiomyopathies, preventing sudden death or cardiac transplantation. It is hoped that further understanding of molecular genetics of cardiomyopathies could well lead to clinical advances. In particular, future therapeutic approaches will include repurposed molecularly directed drugs, siRNA-based gene silencing, and genome editing [170]. It is hoped that further understanding of molecular genetics of cardiomyopathies could well lead to clinical advances.

## 4. Key Messages

- The American Heart Association describes a classification system that categorizes cardiomyopathy as primary or secondary. In primary cases, the disease process is chiefly confined to the heart. Secondary cardiomyopathy describes conditions in which cardiac involvement occurs as part of a systemic condition. This classification system is imperfect, and there is often overlap.
- Hypertrophic cardiomyopathy (HCM) is the most common primary cardiomyopathy, with a prevalence of 1:500 persons. Many patients with HCM are asymptomatic and are diagnosed during family screening,
- Dilated cardiomyopathy (DCM) has a prevalence of 1:2.500 and is the leading indication for heart transplantation. DCM can occur at any age, but is most common in patients 40 to 59 years of age. Symptom’s characteristic of DCM includes arrhythmias and thromboembolic events. Pathogenic or likely pathogenic variants were found in eight genes, (20%) of which are not included in a standard commercially available dilated cardiomyopathy panel.
- LV involvement in ARVC is characterized by clinical and cardiac magnetic resonance features which differ from those seen in DCM. The most distinctive feature of ARVC-LV phenotype is the large amount of fibrosis, which directly and negatively impacts the LV systolic function.
- Restrictive cardiomyopathy is the least common of the major cardiomyopathies, representing 2% to 5% of cases. The restrictive category includes many underlying etiologies and is defined by physiologic function rather than anatomy. Genetic-based RCM might be induced by mutations in genes of nonsarcomeric, sarcomeric, and sarcomere-associated proteins.
- Takotsubo cardiomyopathy is defined as an abrupt onset of left ventricular dysfunction in response to severe emotional or physiologic stress. Postmenopausal women are most commonly affected.

## Data Availability

The data presented in this study are available on request from the corresponding author.
